# Global analysis of *Saccharomyces cerevisiae* growth in mucin

**DOI:** 10.1093/g3journal/jkab294

**Published:** 2021-08-18

**Authors:** Kevin Mercurio, Dylan Singh, Elizabeth Walden, Kristin Baetz

**Affiliations:** Department of Biochemistry, Microbiology and Immunology, Ottawa Institute of Systems Biology, University of Ottawa, Ottawa, ON K1H 8M5, Canada; Department of Biochemistry, Microbiology and Immunology, Ottawa Institute of Systems Biology, University of Ottawa, Ottawa, ON K1H 8M5, Canada; Department of Biochemistry, Microbiology and Immunology, Ottawa Institute of Systems Biology, University of Ottawa, Ottawa, ON K1H 8M5, Canada; Department of Biochemistry, Microbiology and Immunology, Ottawa Institute of Systems Biology, University of Ottawa, Ottawa, ON K1H 8M5, Canada

**Keywords:** *Saccharomyces cerevisiae*, mucin, metabolism, mitochondria, chemical genomics, transcriptome

## Abstract

Metagenomic profiling of the human gut microbiome has discovered DNA from dietary yeasts like *Saccharomyces cerevisiae*. However, it is unknown if the *S. cerevisiae* detected by common metagenomic methods are from dead dietary sources, or from live *S. cerevisiae* colonizing the gut similar to their close relative *Candida albicans*. While *S. cerevisiae* can adapt to minimal oxygen and acidic environments, it has not been explored whether this yeast can metabolize mucin, the large, gel-forming, highly glycosylated proteins representing a major source of carbon in the gut mucosa. We reveal that *S. cerevisiae* can utilize mucin as their main carbon source, as well as perform both a transcriptome analysis and a chemogenomic screen to identify biological pathways required for this yeast to grow optimally in mucin. In total, 739 genes demonstrate significant differential expression in mucin culture, and deletion of 21 genes impact growth in mucin. Both screens suggest that mitochondrial function is required for proper growth in mucin, and through secondary assays we determine that mucin exposure induces mitogenesis and cellular respiration. We further show that deletion of an uncharacterized ORF, *YCR095W-A*, led to dysfunction in mitochondrial morphology and oxygen consumption in mucin. Finally, we demonstrate that Yps7, an aspartyl protease and homolog to mucin-degrading proteins in *C. albicans*, is important for growth on mucin. Collectively, our work serves as the initial step toward establishing how this common dietary fungus can survive in the mucus environment of the human gut.

## Introduction

The human gut microbiome is a vast community of microorganisms that are involved in the homeostasis of our gut physiology, metabolism and nutrient uptake, immune system functionality, and pathogenesis (Sommer and Bäckhed 2013; Shreiner *et al.* 2015). Although mainly focusing on bacteria, the development of high-throughput sequencing-based metagenomics has led to research on the fungal community (*i.e.*, mycobiome) within the gut (Mukherjee *et al.* 2015; Huseyin *et al.* 2017). Currently, and unlike the bacteriome, there is no consensus on a core mycobiome, but recent studies have aimed at determining what fungal species healthy and diseased individuals possess in the gut (McCusker *et al.* 1994; Ott *et al.* 2008; Scanlan and Marchesi 2008; Chen *et al.* 2011; Gouba *et al.* 2014; Li *et al.* 2014; Chehoud *et al.* 2015; Hallen-Adams *et al.* 2015; Rodríguez *et al.* 2015; Strope *et al.* 2015; Hoarau *et al.* 2016; Liguori *et al.* 2016; Strati *et al.* 2016; Hamad *et al.* 2017; Nash *et al.* 2017; Sokol *et al.* 2017; Nagpal *et al.* 2020). However, most metagenomic studies lack culture-dependent analyses and cannot differentiate between live fungi residing in the gut or dead fungal remnants from diet and the environment as the source for detection (Sam *et al.* 2017).

Identifying gut fungal residents is crucial as they are believed to have an important role in human health. In a 2017 review of gut microbiome publications, only 15 fungal species were reported in five or more studies (Hallen-Adams and Suhr 2017). The most commonly reported were the closely related *Candida* spp. and *Saccharomyces* spp. *Candida albicans* is the most frequently studied fungus regarding the impact of the mycobiome on human health due to its virulent ability in immunocompromised individuals (Hall and Noverr 2017; Galloway-Peña and Kontoyiannis 2020). Despite being one of the most common dietary fungi, *Saccharomyces cerevisiae* is often overlooked during discussions of microbial impact within the human gut. For example, while research has been conducted on the subspecies *Saccharomyces boulardii* as a probiotic additive in the food industry, as well as a medical treatment for diarrhea, pathogenic infection, and inflammatory bowel disease (Kelesidis and Pothoulakis 2012), very little is known about the impact *S. cerevisiae* has on human health. *S. cerevisiae* is known to reduce symptoms of colitis and even overturn viral infection by immunological stimulation in mice and humans (Jawhara *et al.* 2012; Sivignon *et al.* 2015; Jiang *et al.* 2017; Sokol *et al.* 2017). Conversely, *S. cerevisiae* was shown to increase intestinal damage and permeability when inoculated in germ-free mice, due to the enhancement of host purine metabolism and induction of uric acid synthesis (Chiaro *et al.* 2017).

Current high-throughput studies for determining fungal composition have discovered *S. cerevisiae* in stool samples (Ott *et al.* 2008; Chen *et al.* 2011; Chehoud *et al.* 2015; Hallen-Adams *et al.* 2015; Rodríguez *et al.* 2015; Hoarau *et al.* 2016; Strati *et al.* 2016; Hamad *et al.* 2017; Nash *et al.* 2017; Sokol *et al.* 2017), with some studies detecting *S. cerevisiae* from mucosa samples (McCusker *et al.* 1994; Li *et al.* 2014; Strope *et al.* 2015; Liguori *et al.* 2016). Importantly, these findings did not conclude whether the identified genetic material came from colonized cells, or dead/dormant cells passing through the gastrointestinal tract. Yet, for any organism to survive in the gut, they must be able to adapt to gut conditions like body temperature, low-oxygen content, mild acidity, and a mucus-rich environment. While *S. cerevisiae* is known to adapt to low oxygen and acidic conditions, it is not known whether the fungus can live off resources found in the gut mucosa.

Lining the epithelium of the gut, the mucosal dual layer system serves to lubricate the passage of food, as well as to protect host cells from intestinal damage and pathogen invasion (Johansson *et al.* 2013). Mucus is composed of water, salts, immunoglobulins, secreted proteins, and mucin (Bansil *et al.* 1995), the latter constituting an abundant supply of carbohydrates found in the gut. In fact, estimates for these large, gel-forming, highly glycosylated proteins suggest that 80% of the total mass of mucins are carbohydrates (Kesimer and Sheehan 2012; Johansson *et al.* 2013; Tailford *et al.* 2015). Upon initial glycosylation, 10 or more sugar moieties can be further attached, leading to a large heterogeneric network of N-acetyl galatacosamine, N-acetyl glucosamine, galactose, fucose, and sialic acid (Rhodes 1989).

Residential fungi like *C. albicans* have a family of 10 secreted aspartyl proteases (SAPs) that allow them to break down mucin as an energy source and live within mucus niches of the gut (Colina *et al.* 1996; De Repentigny *et al.* 2000). Interestingly, *C. albicans* shares 90% of its genome with *S. cerevisiae* (Naglik *et al.* 2003), including six SAP homologs named yapsins. The yapsins that share the most peptide sequence similarity to SAPs, Yps1 and Yps3, are GPI-anchored proteases that are most functional at pH 5-6 (Olsen *et al.* 1999; Gagnon-Arsenault *et al.* 2008; Dubé *et al.* 2015), similar to the average pH of the human colon (Nugent *et al.* 2001). In addition, Yps1, Yps2, and Yps3 are associated with proteolytic activity at the cell surface (Egel‐Mitani *et al.* 1990; Komano and Fuller 1995; Vadaie *et al.* 2008), and various yapsin deletion mutants were differentially susceptible to compounds that targeted the cell wall (Krysan *et al.* 2005). Their potential role in resource collection by degradation or uptake of mucin has yet to be explored.

As a first step to assess if *S. cerevisiae* has the potential to colonize the gut, we investigated whether the laboratory *S. cerevisiae* strain S288C has the ability to utilize mucin as a carbon source. We show that *S. cerevisiae* can use mucin as a carbon source in both solid agar and liquid growth media. To further characterize how *S. cerevisiae* remodels itself in the presence of mucin, we perform both a mucin transcriptome analysis and a chemogenomic screen. Both screens indicate that mitochondrial function is critical for growth in mucin, and through secondary assays we show that mucin induces mitochondrial biogenesis and cellular respiration. We show that the deletion of the uncharacterized ORF *YCR095W-A* leads to slow growth in the presence of mucin likely due to defects in mitochondrial morphology and respiration. Lastly, we demonstrate that the ability of *S. cerevisiae* to utilize mucin in media is dependent on yapsins, especially the uncharacterized Yps7. Collectively, our work serves as the initial step toward establishing how this common dietary fungus could survive in the mucus environment of the human gut.

## Materials and methods

### Yeast strains and media

All strains used in this study are listed in Table 1. Strains were created by either standard mating procedures or by the PCR-mediated gene deletion/insertion technique (Longtine *et al.* 1998), while confirmed via growth on drug selection (G418, NAT, and HYG) or nutrient deprivation, and PCR. Strains taken from the *S.* *cerevisiae* deletion mutant array (DMA) (GE, CAT#YSC1053) and GFP collection (Thermo, CAT#95702) were PCR confirmed. Cells were grown in standard yeast-peptone (YP) medium of 10 g/L yeast extract (Multicell), 20 g/L bacteriological peptone (Multicell), and 0.33 g/L of L-tryptophan (Sigma-Aldrich). Carbon sources were added to media after autoclave sterilization at final concentrations of 2% dextrose (YPD) or 0.5% Type III porcine gastric mucin (YPM) (Sigma-Aldrich, M1778). This concentration of mucin was chosen due to previous work conducted on *C. albicans* (Kavanaugh *et al.* 2014), and mucin stocks were sterilized by autoclave at 121° for 15 min (Terra *et al.* 2010). Agar plates were created using a final concentration of 20 g/L agar (BioShop) added prior to autoclave.

**Table 1:** Strain list

Strain	Genotype	Source
YKB1079/BY4741	**MATa** *his3*Δ*1 leu2*Δ*0 met15*Δ*0 ura3*Δ*0*	Brachmann *et al.* (1997)
YKB1117/BY4743	**MATa/MATα** *his3Δ1/his3Δ1 leu2Δ0/leu2Δ0 LYS2/lys2Δ0 met15Δ0/MET15 ura3Δ0/ura3Δ0*	Brachmann *et al.* (1997)
YKB1118/BY4742	**MATα** *his3*Δ*1 leu2*Δ*0 lys2*Δ*0 ura3*Δ*0*	Brachmann *et al.* (1997)
YKB4828	**MATa** *his3*Δ*1 leu2*Δ*0 met15*Δ*0 ura3*Δ*0*	DMA collection (GE)
	*yps1*Δ::KANMX	
YKB4829	**MATa** *his3*Δ*1 leu2*Δ*0 met15*Δ*0 ura3*Δ*0*	DMA collection (GE)
	*yps3*Δ::KANMX	
YKB4830	**MATa** *his3*Δ*1 leu2*Δ*0 met15*Δ*0 ura3*Δ*0*	DMA collection (GE)
	*yps5*Δ::KANMX	
YKB4831	**MATa** *his3*Δ*1 leu2*Δ*0 met15*Δ*0 ura3*Δ*0*	DMA collection (GE)
	*yps7*Δ::KANMX	
YKB4832	**MATa** *his3*Δ*1 leu2*Δ*0 met15*Δ*0 ura3*Δ*0*	This study
	*yps6*Δ::KANMX	
YKB4835	**MATα** *his3*Δ*1 leu2*Δ*0 lys2*Δ*0 ura3*Δ*0*	This study
	*yps1*Δ::NATMX	
YKB4836	**MATα** *his3*Δ*1 leu2*Δ*0 lys2*Δ*0 ura3*Δ*0*	This study
	*yps3*Δ::NATMX	
YKB4837	**MATα** *his3*Δ*1 leu2*Δ*0 lys2*Δ*0 ura3*Δ*0*	This study
	*yps7*Δ::NATMX	
YKB4897	**MATα** *his3*Δ*1 leu2*Δ*0 lys2*Δ*0 ura3*Δ*0*	This study
	*yps1*Δ::NATMX *yps7*Δ::KANMX	
YKB4898	**MATα** *his3*Δ*1 leu2*Δ*0 lys2*Δ*0 ura3*Δ*0*	This study
	*yps3*Δ::NATMX *yps7*Δ::KANMX	
YKB4899	**MATa** *his3*Δ*1 leu2*Δ*0 met15*Δ*0 ura3*Δ*0*	This study
	*yps1*Δ::KANMX *yps3*Δ::NATMX	
YKB4900	**MATa** *his3*Δ*1 leu2*Δ*0 met15*Δ*0 ura3*Δ*0*	This study
	*yps1*Δ::KANMX *yps3*Δ::NATMX *yps7*Δ::HYGMX	
YKB4901	**MATa** *his3*Δ*1 leu2*Δ*0 met15*Δ*0 ura3*Δ*0*	This study
	*YPS1-GFP*::*HIS3*	
YKB4902	**MATa** *his3*Δ*1 leu2*Δ*0 met15*Δ*0 ura3*Δ*0*	Ghaemmaghami *et al.* (2003)
	*YPS3-GFP*::*HIS3*	
YKB4903	**MATa** *his3*Δ*1 leu2*Δ*0 met15*Δ*0 ura3*Δ*0*	Ghaemmaghami *et al.* (2003)
	*YPS7-GFP::HIS3*	
YKB4907	**MATa** *his3*Δ*1 leu2*Δ*0 met15*Δ*0 ura3*Δ*0*	This study
	*CIT1-RFP*::*URA3*	
YKB4908	**MATa** *his3*Δ*1 leu2*Δ*0 met15*Δ*0 ura3*Δ*0*	DMA collection (GE)
	*ccm1*Δ::KANMX	
YKB4912	**MATa** *his3*Δ*1 leu2*Δ*0 met15*Δ*0 ura3*Δ*0*	DMA collection (GE)
	*ycr095w-a*Δ::KANMX	
YKB4916	**MATa** *his3*Δ*1 leu2*Δ*0 met15*Δ*0 ura3*Δ*0*	This study
	*CIT1-RFP*::*URA3 ycr095w-a*Δ::KANMX	
YKB4917	**MATa** *his3*Δ*1 leu2*Δ*0 met15*Δ*0 ura3*Δ*0*	DMA collection (GE)
	*slt2*Δ::KANMX	
YKB4942	**MATa** *his3*Δ*1 leu2*Δ*0 met15*Δ*0 ura3*Δ*0*	This study
	*CIT1*-RFP::*URA3 ccm1*Δ::KANMX	
YKB5015	**MATa** *his3*Δ*1 leu2*Δ*0 met15*Δ*0 ura3*Δ*0*	DMA collection (GE)
	*yps2*Δ::KANMX	
SK1	***MATa/MATα*** *ho: LYS2/ho: LYS2, ura3/ura3, lys2/lys2, leu2: hisG/leu2: hisG, his3::hisG/his3::hisG, trp1::hisG/trp1::hisG*	

### Dot assays

Strains were grown overnight at 30° in YPD. Cultures were then reinoculated into 5 mL of YPD to an OD_600_ of 0.1 and incubated at 30°. Once they reached log phase, cultures were washed twice in YP, and diluted to an OD_600_ of 0.2 in YP. Dot assays were performed as described previously (Walden *et al.* 2020) with some modifications. Briefly, 3 μL of 10-fold serial dilutions (OD_600_ = 0.2, 0.02, 0.002, 0.0002) were spotted on indicated media and incubated for 2–6 days at indicated temperatures. Images of dot assays were taken using the Bio-Rad Chemidoc™ XRS system under EPI-white light illumination and ImageLab software.

### Time course growth and live-cell microscopy

BY4743 cells were grown overnight at 30° in YPD. Cultures were then reinoculated into 50 mL of YPD to an OD_600_ of 0.1 and incubated at 30°. Once they reached log phase, cultures were washed twice in YP and resuspended in 10 mL of YP. Cell suspensions were diluted to an OD_600_ of 0.1 in 50 mL of indicated media and incubated for 6 days at 30°. On each day, 100 μL was aliquoted from each culture for cell counting on a hemocytometer. In addition, 5 mL was aliquoted from each culture, spun down, and resuspended in SC (no dextrose) for brightfield imaging (0.006 s exposure time, 100% gain) using the Leica DMI 6000 fluorescent microscope (Leica Microsystems GmbH, Wetzler, Germany) equipped with a Sutter DG4 light source (Sutter Instruments, CA, USA), Ludl emission filter wheel with Chroma band pass emission filters (Ludl Electronic Products Ltd., NY, USA) and the Hamamatsu Orca AG camera (Hamamatsu Photonics, Herrsching am Ammersee, Germany).

### RNA extraction

BY4743 cells were grown in triplicate in 50 mL of YPD at 30° to log phase. Cultures were then washed twice in YP and resuspended in 10 mL of YP. Cell suspensions were diluted to an OD_600_ of 0.1 in 100 mL of YP and YPM, and incubated until log phase (24 h) at 30°. Cell counts were performed as previously described in order to normalize each sample at harvest. Cells were washed in dH_2_O, transferred to 2 mL screw-capped tubes and pellets were flash frozen in liquid nitrogen. Pellets were resuspended in 200 μL of breaking buffer (2% Triton X-100, 1% SDS, 100 mM NaCl, 100 mM Tris-HCl [pH 8.0], 1 mM EDTA) and 200 μL of a phenol: chloroform: isoamyl alcohol mixture (125:24:1, pH 4.5) (Thermo, CAT#AM9720) was added to each sample. Glass beads of size 0.5 mm (BioSpec, CAT#11079105) were added to just under the sample meniscus and tubes were vortexed for 3.5 min. Samples were centrifuged at maximum speed for 25 min at 4°, and the aqueous phase was aliquoted into a sterile 1.5 mL microcentrifuge tube. Total RNA was ethanol precipitated out of solution and resuspended in nuclease-free water. Three DNase treatments using DNaseI (Promega, CAT#M6101) for 1 h at 37° were performed to remove genomic DNA, and subsequently purified using the RNA Clean and Concentrator-25 kit (ZymoResearch, CAT#R1017). RNA integrity was assessed by gel electrophoresis using a 0.8% agarose gel. RNA purity was assessed through two different ways: (1) the Thermo Scientific Nanodrop 2000 spectrophotometer and (2) the Agilent Bioanalyzer and RNA 6000 Nano quantification kit (Agilent, CAT#5067-1511), the latter used specifically for RNA sequencing.

### RNA sequencing

New England Biolabs rRNA-depleted (Yeast RiboZero) stranded library preparation and RNA sequencing using the Illumina NovaSeq6000 S2 PE100 were conducted by the Genome Quebec Sequencing Centre at McGill University. Reads were aligned to the *S. cerevisiae* genome assembly R64-1-1 using STAR (Dobin *et al.* 2013) and differential expression analysis was performed using DESeq2 (Love *et al.* 2014) by the Canadian Centre for Computational Genomics (C3G). Transcripts with a false discovery rate (FDR) adjusted *P*-value ≤ 0.01 were considered to be differentially expressed. In brief, reads were trimmed using Trimmomatic (Bolger *et al.* 2014) from the 3’-end and filtered by setting a PHRED score cutoff of at least 30 and also have a length of at least 32 bp. Estimated transcript abundances via the metric fragments per kilobase of exon per million fragments mapped (FPKM) was performed using Cufflinks (Roberts *et al.* 2011). Gene ontology (GO) analysis was done using DAVIDv6.8 (Huang *et al.* 2009).

### Genome-wide chemogenomic screen

The *S. cerevisiae* MATa yeast DMA (Winzeler *et al.* 1999), with approximately 4200 strains, was arrayed in duplicate on YPD agar (condensed to 1536 colonies per plate) supplemented with G418 using a Singer RoTor HDA. Using these condensed plates, colonies were pinned onto triplicate YP and YPM agar plates and incubated at 30°. The number of days for incubation depended on when each condensed plate had the lowest difference in average colony size between media, leading to a range of 2–4 days. Images were taken using the Bio-Rad Chemidoc™ XRS system under EPI-white light illumination and growth was assessed using SGAtools (Wagih *et al.* 2013). Colonies were aligned to gene names using R Suite. Average growth scores on each medium were assessed by comparing strain growth on YPM to YP, and calculated ratios for every strain were ranked on whether they demonstrated increased growth on mucin (*i.e.*, positive impact and larger colonies) or decreased growth on mucin (*i.e.*, negative impact and smaller colonies). Ratios were obtained through two different approaches: (1) by comparing average colony sizes of each strain from YPM to YP, and (2) by comparing each individual pinned colony of each strain from YPM to YP. By combining the top 30 strains from the positive impact group and negative impact group through each approach, these hits were confirmed by conducting dot assays on YP and YPM. Strains were subsequently categorized into two groups: (1) YPM > YP and (2) YPM < YP.

### Mitochondrial morphology and abundance

Cit1-RFP strains were grown overnight at 30° in YPD. Cultures were then reinoculated into 50 mL of YP, YPM and YPD to an OD_600_ of 0.1 and incubated at 30°. After 24 h, 5 mL of culture was harvested and cells were resuspended in SC (no dextrose) for brightfield (0.006 s exposure time, 100% gain) and RFP fluorescence (0.5 s, 100% gain) imaging as described previously. Z-stacked images were taken at 0.2 μM steps for a total of 31 planes, and were taken at multiple fields of view in order to capture at least 50 cells per sample on the 63X oil-immersion objective. RFP fluorescence was quantified using the TCCF method (McCloy *et al.* 2014), normalized to WT cell fluorescence in the same medium.

### Oxygen consumption rate assay

Oxygen consumption rate (OCR) assays were performed with a Seahorse XFe96 instrument (Agilent) as described previously (Walden *et al.* 2020) with some modifications. Briefly, overnight cultures grown in YPD were washed in YP, reinoculated into 120 mL of YP or YPM to an OD_600_ of 0.1, and incubated at 30° for 24 h. To make 2% ethanol supplemented YP (YPE) cultures, overnight strains were reinoculated into 50 mL of YPE to an OD_600_ of 0.1 and incubated for 6 h on the day of the experiment. Poly-L-lysine (Sigma, CAT#25988-63-0) was added to the Agilent Seahorse XF96 plates (CAT#101085-004) by pipetting 30 μL of 0.1 mg/mL into each well and incubating on a rocking platform for 5 min. Plates were allowed to dry for 2 h prior to the experiment. Sensor cartridges were incubated with calibrant at 37° for 2 h as well. After incubation, cultures were split into three falcon tubes (40 mL each), and resuspended into minimal media (0.167% YNB, 0.5% ammonium sulfate). Cell counting was performed to determine cell concentration, and cell suspensions were diluted such that each well containing 180 μL of suspension had 5 × 10^5^ cells. The plate contained three technical replicates and six blanks per medium. The plate was centrifuged at 500 rpm for 3 min and incubated at 30° for 30 min. To stop mitochondrial oxygen consumption during the assay, 0.05% sodium azide (Srikumar *et al.* 2013) was added to the calibrated Seahorse XFe96 sensor cartridge before loading. The OCR was measured three times each, before and after the injection of sodium azide, over 3 min with 1.5 min of mixing in between measurements.

### qRT-PCR

RNA samples were converted to cDNA using the iSCRIPT cDNA synthesis kit (Bio-Rad, CAT#170890). All primers used for qPCR were obtained from ThermoFisher and evaluated for their efficiency by conducting qPCR on pooled cDNA samples. Primers are as follows: (1) *BAR1* F: 5′-AGGAGATGTATTACGCAACA-3′, R: 5′-GGTAAGCAGAAGGGATTGCT-3′; (2) *YPS1* F: 5′-CATCGCAGGTTCTCGGTAAG-3′, R: 5′-CTAGCGAGTCCCCGTAAAGC-3′; (3) *YPS2* F: 5′-GATGATTACGAGCTGGTGGA-3′, R: 5′-TGTCGACAAGCACAGTAACT-3′; (4) *YPS3* F: 5′-AGCAGTCTTAACTAGTCCGG-3′, R: 5′-TCGATCTCTTGCTGAGTTCA-3′; (5) *YPS5* F: 5′-GCTGACATTGCCTATTGCAA-3′, R: 5′-GAGGTGGTAGTAGAACGAGG-3′; (6) *YPS6* F: 5′-GCATCTTGTTTGGTGCAGTG-3′, R: 5′-ATCCCAGGATTTGAGCCAAG-3′; (7) *YPS7* F: 5′-GCAAAGTCTGGAACCTCTTC-3′; R: 5′-GTTGACCGGGAGTGCCAAAT-3′. Serial dilutions of 1:5 were used to determine the most optimal annealing temperature for each primer set. qPCR was performed using the SsoFast™ EvaGreen^®^ Supermix (Bio-Rad, CAT#172-5201) and conducted on the BioRad CFX-96 using the standard two-step annealing procedure: 95° for 3 min followed by 40 cycles of 95° for 10 s, and 57° for 10 s. Cycle threshold (CT) values were obtained via the BioRad CFX Manager software and used for analysis. Fold changes were calculated using the ΔΔCT method (Livak and Schmittgen 2001) with the reference gene *BAR1* and normalized to gene expression levels in YP. Three biological replicates and three technical replicates were used for each sample.

### Statistical analysis

To assess statistical significance between two independent groups, two-tailed unpaired *t*-tests were performed. In experiments with three or more independent groups, two-way ANOVA with Tukey’s multiple comparisons tests were conducted. Standard deviations were calculated for at least three experimental replicates. All statistical analyses were done using the GraphPad Prism 8 software (GraphPad Software Inc., La Jolla, CA, USA).

## Results

### *Saccharomyces cerevisiae* can utilize mucin as a carbon source but reduces cell size

**Figure 1:**
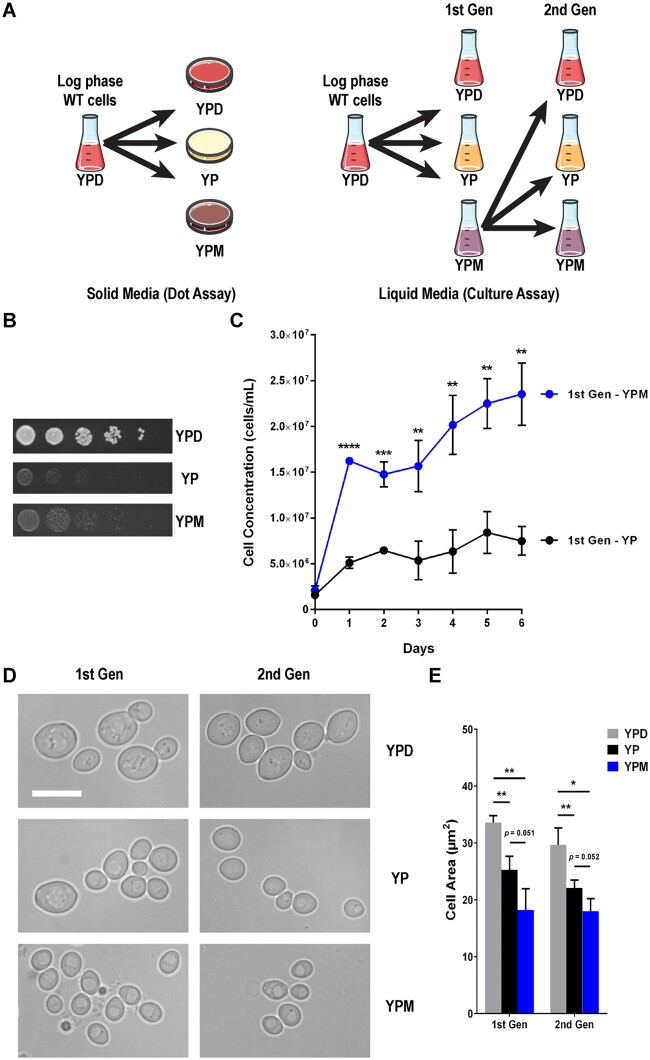
*Saccharomyces cerevisiae* can metabolize mucin as a carbon source but reduces cell size. (A) Flow diagram of yeast growth in solid and liquid mucin media. (B) Mucin did not hinder growth on solid media. WT (YKB1117) cells were grown to log phase in YPD, washed in YP and diluted to a final OD_600_ of 0.2. Four 10-fold serial dilutions were spotted onto YPD, YP, and YPM agar plates. Plates were incubated for 2 days at 30° and are representative of three biological replicates. (C) *S. cerevisiae* grew in mucin liquid culture. WT cells were grown to log phase in YPD, washed in YP and reinoculated into 50 mL of YP (black) and YPM (blue) media (1st Gen). Cultures were incubated for 6 days at 30° and cell concentration was measured via cell counting on a hemocytometer by aliquoting 100 μL of culture every 24 h. (D) Live-cell imaging of *S. cerevisiae* grown in different media. WT cells were grown to log phase in YPD, washed in YP and reinoculated into 50 mL of YPD, YP, and YPM media. First generation cells were incubated for 24 h, at which time 5 mL of each culture was aliquoted for cell harvest. Cell pellets were resuspended in SC (no dextrose) prior to brightfield imaging. After 6 days, cells were harvested, washed in YP and reinoculated into new YPD, YP, and YPM media. Second generation cells were incubated, aliquoted and imaged similarly. Scale bar represents 10 μm. (E) Quantification of first and second generation cell areas for three biological replicates in YPD (grey), YP (black), and YPM (blue) media. All error bars denote standard deviation (SD). *P ≤ 0.05, **P ≤ 0.01, ***P ≤ 0.001, ****P ≤ 0.0001.

As it is unknown whether *S. cerevisiae* can grow in the presence of mucin, we assessed the growth of BY4743 S288C wild type (WT) diploid yeast on solid and liquid media with 0.5% mucin as the only added carbon source to standard YP medium (Figure 1A)*.* Though YP agar plates supported some growth of WT yeast, likely due to the limited carbon available in the forms of free amino acids and short peptides found in yeast extract and peptone, growth on agar plates containing mucin (YPM) was not hindered and may have been improved (Figure 1B). Similarly, cells had significantly better growth in YPM compared to cells grown in YP liquid culture (Figure 1C). As *C. albicans* cells cultured in mucin media become pseudohyphal (Kavanaugh *et al.* 2014), we also assess morphology. As pseudohyphae formation is defective in *S. cerevisiae* S288C due to a mutation in *FLO8* (Liu *et al.* 1996), as expected the *S. cerevisiae* BY4743 cells did not form pseudohyphae when cultured in mucin (Figure 1D), but a pseudohyphae competent SK1 strain does when cultured in YPM (Supplementary Figure S1). While *S. cerevisiae* BY4743 mucin culture could not form pseudohyphae, YPM cultured cells were significantly smaller than cells cultured in YPD and YP by 1.8-fold and 1.4-fold, respectively (Figure 1, D and E). To determine whether the decrease in cell size was due to genetic mutations acquired upon mucin culturing, first generation YPM cells were reinoculated into YPD (second generation) and they reverted back to a normal size. This indicates the decrease in cell size upon culturing in YPM is not permanent and does not reflect a mutation. Taken together, this suggests that BY4743 cells are remodeling their metabolism and morphology in the presence of mucin.

### RNA sequencing demonstrates drastic transcriptome remodeling during growth in mucin and the importance of mitochondrial-associated genes

To assess in an unbiased manner the cellular remodeling that must occur and to identify the cellular processes required for growth in mucin, we compared the transcriptomes of BY4743 cells grown in the absence or presence of mucin. As we wanted to identify genes and cellular processes that were specifically remodeled upon growth in mucin media and not just for growth in media with poor or no added carbon source, we opted to compare transcriptomes from cells grown in YP and YPM for 24 h. Each sample had >90% read alignment to the *S. cerevisiae* genome assembly R64-1-1, accounting for approximately 5000 genes. Principal component analysis demonstrated clustering of sample replicates and variance can be mainly attributed to sample group (Supplementary Figure S2A). In total, 739 genes showed significant differential expression comparing cells in YPM to cells in YP with a fold change of two or more, and a FDR adjusted *P*-value ≤ 0.01 (Supplementary Figure S2B and File S1). With nearly 15% of the genes detected in the analysis significantly remodeled, 235 genes were upregulated and 504 genes were downregulated suggesting that growth in mucin requires a dramatic remodeling of the transcriptome and cellular pathways.

**Figure 2:**
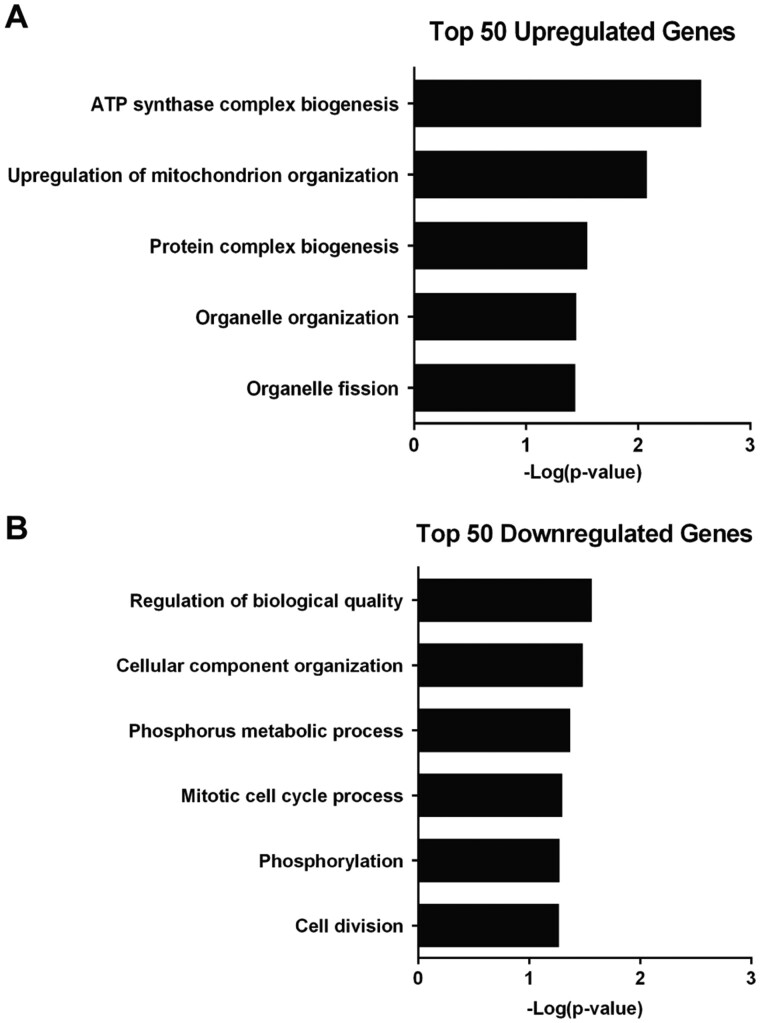
GO analysis for upregulated and downregulated genes is enriched for gene products involved in mitochondrial function and the cell cycle. (A) Functional enrichment for top 50 upregulated genes in YPM compared to YP. (B) Functional enrichment for top 50 downregulated genes in YPM compared to YP. Genes were organized based on biological process using DAVIDv6.8 and plotted with a threshold *P*-value of ≤ 0.05.

To identify pathways modulated during growth in mucin, we chose to assess the top 50 upregulated (Table 2) and downregulated (Table 3) genes for GO analyses using DAVIDv6.8 (Huang *et al.* 2009). Each list of genes contains approximately 20% that have gene products of unknown function. Among the upregulated genes, there was an enrichment for gene products involved in mitochondrial function including ATP synthase biogenesis (*P *=* *0.00273) and positive regulation of mitochondrion organization (*P *=* *0.00837) (Figure 2A). Another GO category enriched for was organelle fission (*P *=* *0.0363), a common feature of the mitochondrion. Among the downregulated genes, there was an enrichment for gene products involved in the mitotic cell cycle process (*P *=* *0.0504) and cell division (*P *=* *0.0541) (Figure 2B), including a 15-fold reduction in expression of the essential transcriptional activator *NDD1*. Ndd1 is critical for transcription of late-S-phase genes *CLB1*, *CLB2* and *SWI5*, and its depletion leads to a mitotic arrest of cells with 2 N DNA and an undivided nucleus (Loy *et al.* 1999). Overall, this suggests that cells require increased mitochondrial function for mucin metabolism and that progression through the cell cycle is likely affected in mucin conditions.

**Table 2:** Top 50 upregulated genes in YPM compared to YP ranked by fold change

Rank	Systematic name	Gene name	Fold change	*P*-value	FDR
1	YJR068W	*RFC2*	15.89	1.30e-09	9.74e-07
2	YPL270W	*MDL2*	15.12	1.09e-07	7.74e-06
3	YHL026C	*YHL026C*	14.88	3.78e-08	4.06e-06
4	YPL160W	*CDC60*	13.06	6.26e-07	2.35e-05
5	YPL283C	*YRF1-7*	11.19	1.32e-05	1.64e-04
6	YNL071W	*LAT1*	9.43	2.41e-06	5.53e-05
7	YDR278C	*YDR278C*	9.20	7.06e-04	3.02e-03
8	tQ(CUG)M	*CDC65*	6.40	1.73e-05	1.96e-04
9	YLR315W	*NKP2*	5.89	9.31e-06	1.32e-04
10	YDR350C	*ATP22*	5.78	5.73e-08	5.44e-06
11	YPR091C	*NVJ2*	5.56	5.31e-05	4.23e-04
12	YDR203W	*YDR203W*	5.56	2.26e-05	2.34e-04
13	YPL200W	*CSM4*	5.53	7.04e-09	1.97e-06
14	YER168C	*CCA1*	5.42	9.13e-07	2.98e-05
15	YLR133W	*CKI1*	5.35	1.09e-07	7.74e-06
16	YDR492W	*IZH1*	5.26	1.45e-06	3.91e-05
17	YDL118W	*YDL118W*	5.19	3.04e-06	6.46e-05
18	YOR343C	*YOR343C*	5.08	3.21e-10	4.50e-07
19	YDR014W-A	*HED1*	4.86	1.63e-08	3.29e-06
20	YGL171W	*ROK1*	4.49	2.05e-05	2.18e-04
21	YNR020C	*ATP23*	4.46	9.13e-08	6.90e-06
22	YDL237W	*AIM6*	4.45	7.27e-05	5.27e-04
23	YHL007C	*STE20*	4.31	2.29e-07	1.23e-05
24	YKL187C	*FAT3*	4.21	1.33e-07	8.86e-06
25	YJR072C	*NPA3*	4.20	5.28e-07	2.11e-05
26	YDR301W	*CFT1*	4.18	1.99e-07	1.12e-05
27	YER154W	*OXA1*	4.12	9.84e-07	3.06e-05
28	YLR203C	*MSS51*	4.11	2.35e-08	3.60e-06
29	YFR042W	*KEG1*	4.10	2.35e-06	5.42e-05
30	YPL161C	*BEM4*	4.05	4.37e-06	8.32e-05
31	YPR143W	*RRP15*	4.04	7.61e-06	1.17e-04
32	YFR041C	*ERJ5*	3.98	9.55e-08	7.06e-06
33	YLR397C	*AFG2*	3.89	2.25e-08	3.60e-06
34	YDR320W-B	*YDR320W-B*	3.78	1.38e-06	3.81e-05
35	YBR260C	*RGD1*	3.70	2.70e-05	2.68e-04
36	YIL065C	*FIS1*	3.68	3.48e-05	3.17e-04
37	YIL021W	*RPB3*	3.67	3.11e-04	1.59e-03
38	YHR150W	*PEX28*	3.65	6.30e-06	1.03e-04
39	YOR268C	*YOR268C*	3.63	2.69e-09	1.13e-06
40	YJR094W-A	*RPL43B*	3.60	4.54e-06	8.52e-05
41	YGL066W	*SGF73*	3.58	1.11e-05	1.49e-04
42	YOL099C	*YOL099C*	3.58	4.83e-04	2.24e-03
43	YLR190W	*MMR1*	3.57	3.46e-08	4.01e-06
44	YNL312W	*RFA2*	3.55	6.50e-06	1.05e-04
45	YLR447C	*VMA6*	3.53	4.19e-09	1.57e-06
46	YMR138W	*CIN4*	3.52	6.06e-06	1.00e-04
47	YER055C	*HIS1*	3.51	2.30e-03	7.73e-03
48	YKR023W	*RQT4*	3.44	1.40e-05	1.71e-04
49	YLR191W	*PEX13*	3.44	1.86e-07	1.08e-05
50	YOR245C	*DGA1*	3.41	5.54e-06	9.64e-05

**Table 3:** Top 50 downregulated genes in YPM compared to YP ranked by fold change

Rank	Systematic name	Gene name	Fold change	*P*-value	FDR
1	YPL227C	*ALG5*	−21.47	1.05e-09	8.80e-07
2	YOR372C	*NDD1*	−15.73	3.84e-11	1.29e-07
3	YNL273W	*TOF1*	−14.46	3.52e-11	1.29e-07
4	YNL047C	*SLM2*	−13.73	4.19e-10	4.50e-07
5	YHR214W	*YHR214W*	−13.05	3.78e-08	4.06e-06
6	YLR164W	*SHH4*	−11.85	2.18e-08	3.60e-06
7	YGL248W	*PDE1*	−11.37	4.32e-04	2.05e-03
8	YBR056W-A	*MNC1*	−10.78	2.93e-10	4.50e-07
9	YHR107C	*CDC12*	−9.66	3.37e-10	4.50e-07
10	YHR206W	*SKN7*	−9.47	1.36e-08	3.25e-06
11	YLR385C	*SWC7*	−9.31	1.97e-09	1.02e-06
12	tE(UUC)Q	*tE(UUC)Q*	−8.53	1.91e-09	1.02e-06
13	YMR155W	*YMR155W*	−8.21	1.73e-07	1.04e-05
14	snR18	*snR18*	−8.02	2.18e-08	3.60e-06
15	YJR105W	*ADO1*	−7.96	2.92e-07	1.45e-05
16	YBR131W	*CCZ1*	−7.94	7.46e-08	6.12e-06
17	YGL253W	*HXK2*	−7.89	6.13e-07	2.33e-05
18	YGR033C	*TIM21*	−7.88	7.36e-07	2.64e-05
19	YAR020C	*PAU7*	−7.80	2.65e-05	2.65e-04
20	YNR018W	*RCF2*	−7.79	1.80e-07	1.05e-05
21	YIL051C	*MMF1*	−7.59	3.62e-06	7.28e-05
22	YOR079C	*ATX2*	−7.46	3.75e-05	3.33e-04
23	YFL060C	*SNO3*	−7.45	1.61e-09	1.02e-06
24	YLR294C	*YLR294C*	−7.32	1.43e-07	9.25e-06
25	YDL097C	*RPN6*	−7.19	5.34e-07	2.12e-05
26	YMR288W	*HSH155*	−6.99	2.11e-09	1.02e-06
27	YHR020W	*YHR020W*	−6.72	1.92e-08	3.60e-06
28	YLR434C	*YLR434C*	−6.53	2.69e-09	1.13e-06
29	YPL233W	*NSL1*	−6.50	7.22e-06	1.13e-04
30	YGL169W	*SUA5*	−6.36	5.78e-08	5.44e-06
31	YML070W	*DAK1*	−6.34	1.14e-04	7.44e-04
32	YMR238W	*DFG5*	−6.30	7.40e-06	1.15e-04
33	YML057W	*CMP2*	−6.30	1.58e-05	1.86e-04
34	YLR086W	*SMC4*	−6.29	4.68e-10	4.50e-07
35	YMR006C	*PLB2*	−6.07	2.44e-08	3.60e-06
36	YHR031C	*RRM3*	−6.04	4.61e-04	2.16e-03
37	YGL068W	*MNP1*	−6.03	1.50e-08	3.25e-06
38	YGR224W	*AZR1*	−5.94	3.31e-08	3.97e-06
39	YKL122C	*SRP21*	−5.80	1.62e-07	1.00e-05
40	YMR218C	*TRS130*	−5.70	3.03e-08	3.71e-06
41	YKL193C	*SDS22*	−5.65	9.38e-09	2.52e-06
42	YNR042W	*YNR042W*	−5.56	7.91e-06	1.20e-04
43	YOL155C	*HPF1*	−5.52	6.58e-08	5.70e-06
44	YIL151C	*ESL1*	−5.48	3.39e-05	3.14e-04
45	YGL174W	*BUD13*	−5.32	1.32e-04	8.36e-04
46	tE(UUC)M	*tE(UUC)M*	−5.26	2.42e-08	3.60e-06
47	YKL150W	*MCR1*	−5.22	1.20e-07	8.20e-06
48	YHR214W-A	*YHR214W-A*	−5.19	8.85e-05	6.10e-04
49	YBR146W	*MRPS9*	−5.15	5.18e-09	1.83e-06
50	YFR019W	*FAB1*	−5.11	1.36e-06	3.78e-05

### Chemogenomic screen identifies biological processes important for growth on mucin including mitochondrial function

**Figure 3:**
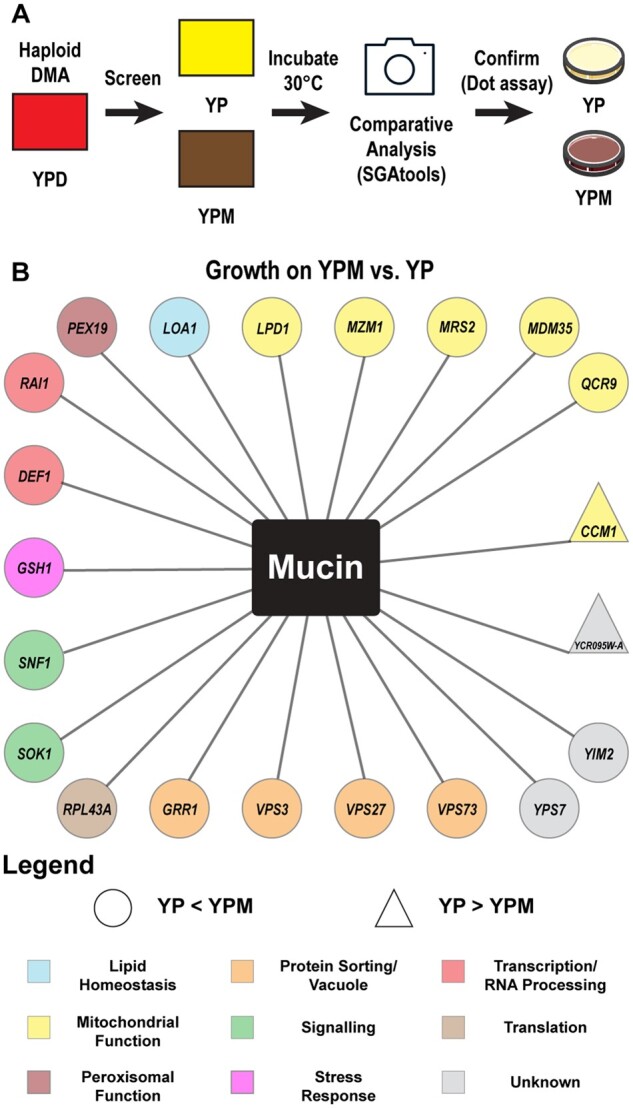
Mucin chemogenomic profile for *S. cerevisiae*. (A) Flow diagram of mucin screen. Comparative analysis was conducted using growth scores obtained via SGAtools for each strain on YP and YPM. YPM/YP score ratios obtained for each strain were calculated and ranked. Confirmation was conducted for the highest (positive impact deletion) and lowest (negative impact deletion) ratios by comparing growth via dot assays. (B) Deletion mutants identified and confirmed in the screen have been color coded based on biological process. Shapes indicate whether gene deletion resulted in a positive impact (grew better on YPM than on YP, YP < YPM, ○) or negative impact (grew worse on YPM than on YP, YP > YPM, Δ).

To complement the transcriptome analysis, we screened the *S. cerevisiae* DMA (Winzeler *et al.* 1999) for genes that specifically modulate growth on mucin when deleted (Figure 3A). To eliminate deletion mutants with defects for growth on poor carbon sources, the DMA was screened in triplicate on both YP and YPM, and all hits were subsequently confirmed by conducting serial dot assays. Twenty-one deletion mutants were confirmed in the screen (Figure 3B). There were 19 deletion mutants that displayed increased growth on mucin (YP growth < YPM growth) and two deletion mutants that displayed decreased growth on mucin (YP growth > YPM growth).

Genes identified in the screen were categorized by biological process using their descriptions listed in the *Saccharomyces* Genome Database (https://www.yeastgenome.org/). Deletion mutants that displayed improved growth on YPM compared to their growth on YP were implicated in mitochondrial function, protein sorting or vacuolar function, transcription or RNA processing, and signaling, among others. This included the deletion mutant of *SNF1*, the yeast AMPK, which has an established role for growth in poor carbon conditions (Ghillebert *et al.* 2011). *YPS7*, a homolog to mucin degrading SAPs in *C. albicans*, was also identified. Though *yps7*Δ cells growth deficiency is more prominent on YP compared to YPM (YP growth < YPM growth), it is important to note that *yps7*Δ cells also display a growth deficiency on YPM compared to WT cells (see below). This suggests that Yps7 may be important for overall growth in poor conditions when resources are scarce.

The only two deletion mutants that displayed reduced growth on YPM compared to their growth on YP were *CCM1* and *YCR095W-A. CCM1* encodes a mitochondrial 15S rRNA-binding protein that is involved in the stabilization of *COB* and *COX1* pre-mRNAs, both of which encode components of the electron transport chain (Moreno *et al.* 2012). *YCR095W-A* encodes a putative protein of unknown function, however high throughput screens suggest that it may localize to mitochondria and impact mitochondrial function (Breker *et al.* 2014; Stenger *et al.* 2020). Along with the transcriptomic data, this further suggests the importance of mitochondrial function in regards to growth in media with mucin as the main carbon source.

### Mitochondrial fission and biogenesis are induced upon deletion of *CCM1* or *YCR095W-A* during growth in mucin

**Figure 4:**
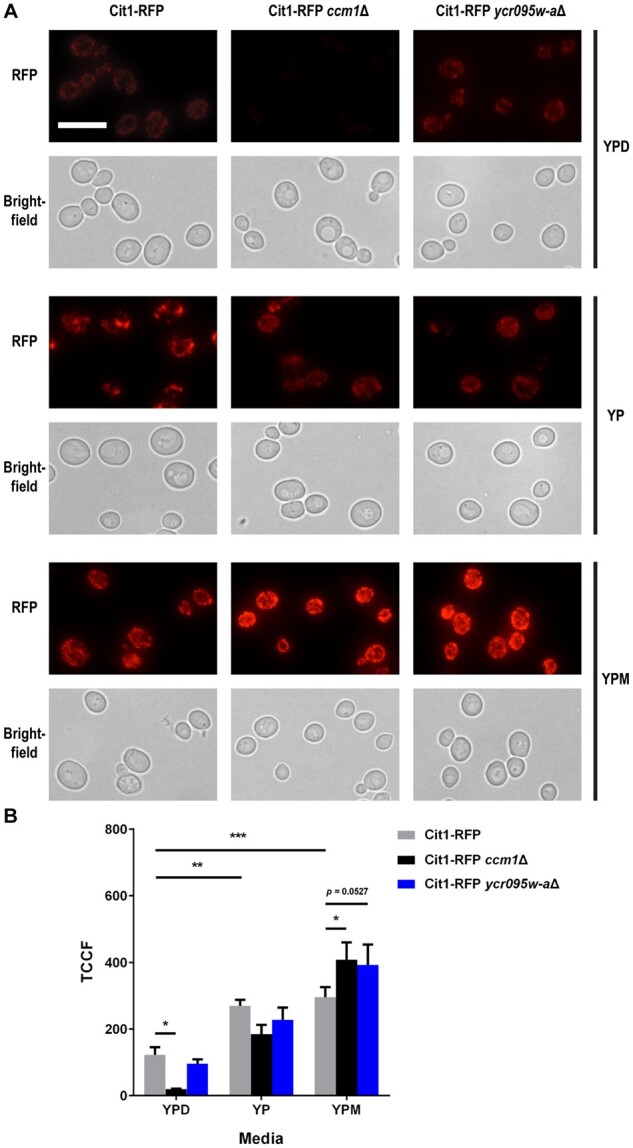
Growth in mucin medium increases Cit1-RFP and changes mitochondrial morphology. (A) Cit1-RFP (YKB4907), Cit1-RFP *ccm1*Δ (YKB4942), and Cit1-RFP *ycr095w-a*Δ (YKB4916) were grown to log phase in YPD, washed in YP and reinoculated into 50 mL of YPD, YP, and YPM media. Cultures were incubated for 24 h, at which time 5 mL of each culture was aliquoted for cell harvest. Cell pellets were resuspended in SC (no dextrose) prior to RFP and brightfield imaging. Scale bar represents 10 μm. (B) Quantification of RFP by total corrected cell fluorescence (TCCF) for three biological replicates in each media, with WT comparisons to *ccm1*Δ and *ycr095w-a*Δ. All error bars denote SD. *P ≤ 0.05, **P ≤ 0.01, ***P ≤ 0.001.

Both the transcriptome and chemical profiling suggest that mitochondria play an important role in the ability of *S. cerevisiae* to utilize mucin. Hence, we further looked at the impact of mucin on mitochondrial morphology. We assessed mitochondrial morphology of WT, *ccm1*Δ and *ycr095w-a*Δ cells that expressed Cit1-RFP. Cit1, or citrate synthase, is the enzyme responsible for the conversion of acetyl coenzyme A into citrate at the beginning of the tricarboxylic acid cycle and is a common mitochondrial marker (Higuchi-Sanabria *et al.* 2016). As expected, the mitochondria in WT cells grown in YPD for 24 h were elongated and localized mainly to the cell periphery, while the mitochondria in WT cells grown for 24 h under the carbon-limiting conditions of YP became shorter, more abundant, and dispersed throughout the cell (Figure 4A). Interestingly, the mitochondria of cells grown in YPM for 24 h seemed to resemble a state in between YP and YPD mitochondria. Furthermore, there was a clear increase in the levels of Cit1-RFP upon incubation in YP and YPM (Figure 4B). *ccm1*Δ cells showed reduced abundance of mitochondria in YPD, while in *ycr095w-a*Δ cells the mitochondria appeared to be similar to the WT state. Upon incubation in YP and YPM, morphological changes were even more enhanced within *ccm1*Δ and *ycr095w-a*Δ cells (Figure 4A). Cit1-RFP fluorescence was significantly higher in *ccm1*Δ and *ycr095w-a*Δ cells compared to WT cells grown in YPM (Figure 4B). Thus, cells respond to the deletion of *CCM1* or *YCR095W-A* as if further starved of carbon sources and induce mitochondrial fission and biogenesis in mucin conditions.

### Mitochondrial function is disrupted upon deletion of *YCR095W-A* during growth in mucin

**Figure 5:**
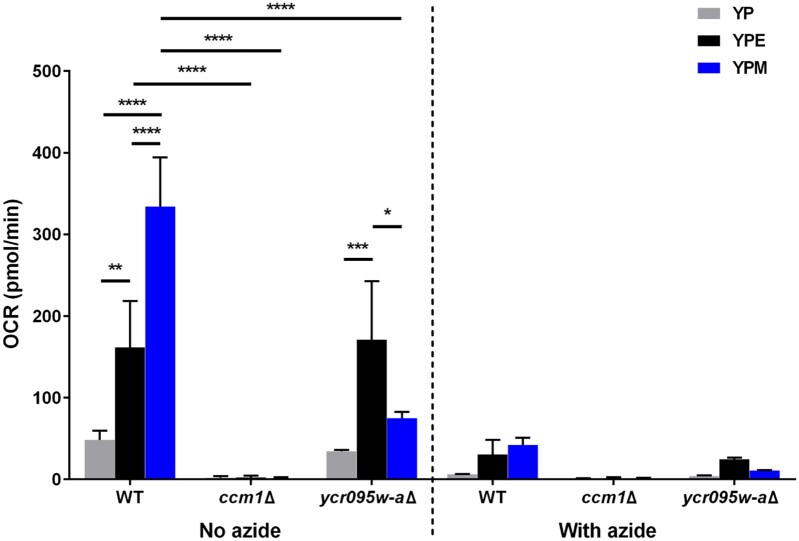
The OCR increases in the presence of mucin and disrupted upon deletion of *CCM1* or *YCR095W-A*. WT (YKB1079), *ccm1*Δ (YKB4908), and *ycr095w-a*Δ (YKB4912) cells were grown to log phase in YPD, washed in YP and reinoculated into 50 mL of YP and YPM media. Cultures were incubated for 24 h, at which time cells were harvested. Cultures for cells grown in ethanol were done on the same day as the assay. Cell pellets were resuspended in YNB medium with water, 2% ethanol or 0.5% mucin, and 5 × 10^5^ cells were aliquoted into designated plate wells. The OCRs were measured via the Agilent Seahorse XFe96 analyzer before and after the addition of 0.05% sodium azide. Error bars denote SD. *P ≤ 0.05, **P ≤ 0.01, ***P ≤ 0.001, ****P ≤ 0.0001.

The changes in morphology suggest there may be changes in mitochondrial function when grown in mucin conditions. Thus, we asked whether there were differences in mitochondrial function between WT, *ccm1*Δ and *ycr095w-a*Δ cells grown for 24 h in YP and YPM. Strains were also grown in YPE for 6 h to ensure that cells were capable of respiration. After incubations, we measured the OCR of strains before and after the injection of 0.05% sodium azide, used to shut down oxygen consumption from mitochondria by binding and inhibiting the function of cytochrome c oxidase of the electron transport chain (Tsubaki and Yoshikawa 1993). We observed that the OCR of WT cells was significantly higher in the presence of an added carbon source, with the highest OCR observed from YPM cells (Figure 5). Despite having Cit1-RFP detectable in mitochondria upon mucin treatment (Figure 4A), the OCR of *ccm1*Δ cells was significantly lower from cells grown in all conditions with an added carbon source compared to the WT. This is not unexpected as null mutants for *CCM1* are known to have dysfunctional mitochondria and therefore have reduced growth on nonfermentable carbon sources (Puchta *et al.* 2010). In contrast, the OCR of *ycr095w-a*Δ cells was only significantly lower than WT cells from YPM populations. This suggests that the product of *YCR095W-A* has a distinct impact on the mitochondria and overall growth when mucin is the main carbon source.

### The aspartyl protease Yps7 is important for *S. cerevisiae* growth on mucin

**Figure 6:**
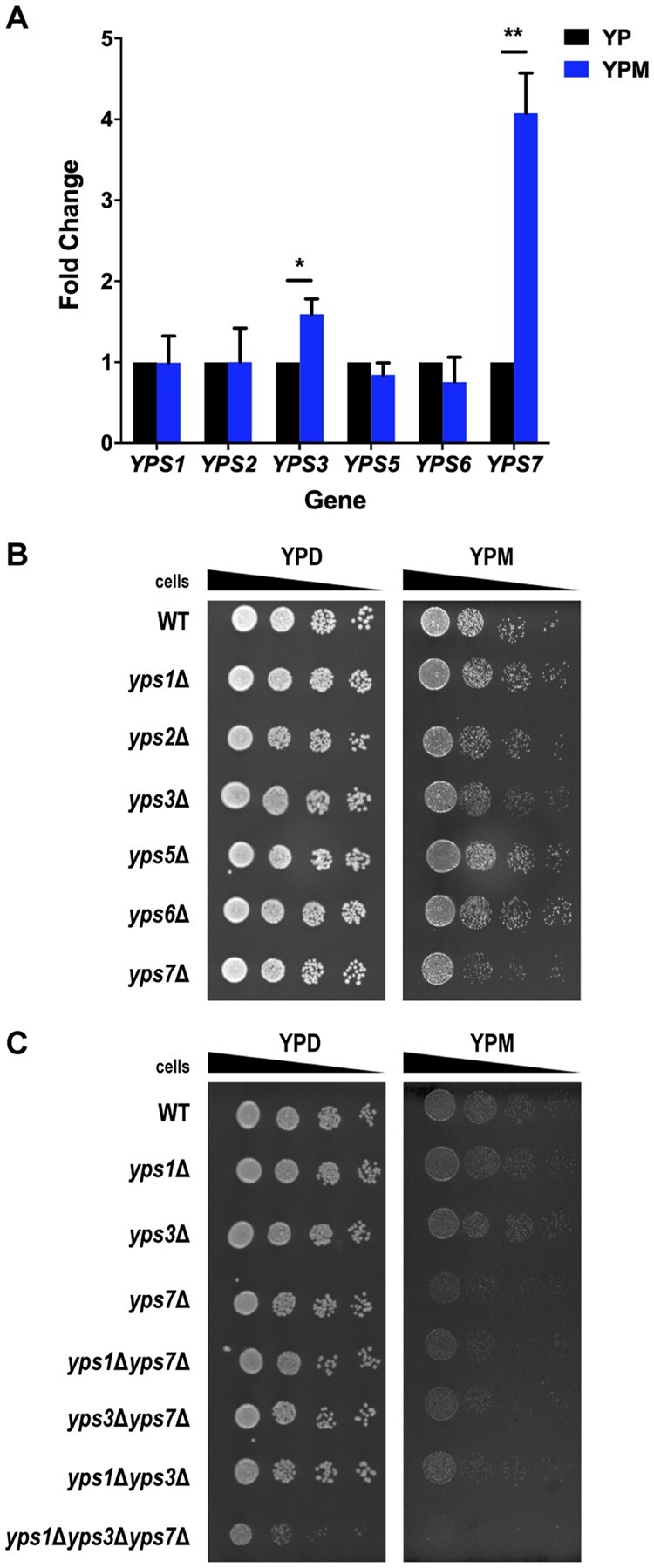
*YPS7* gene expression is induced upon mucin exposure and *yps7*Δ cells display reduced growth on mucin. (A) WT (YKB1117) cells were grown to log phase in YPD, washed in YP and reinoculated into 50 mL of YP (black) and YPM (blue) media. Cultures were incubated for 24 h at 30° prior to cell harvest and normalized to the lowest concentrated culture. Cells were then lysed via bead beating and RNA was extracted via the phenol: chloroform: isoamyl alcohol method. RNA concentration and integrity were assessed via nanodrop and gel electrophoresis. qRT-PCR was conducted using EvaGreen and fold changes were analyzed with the ΔΔCT method normalized to YP levels. All error bars denote SD. *P ≤ 0.05, **P ≤ 0.01. (B) WT (YKB1079), along with *yps1*Δ (YKB4828), *yps2*Δ (YKB5015), *yps3*Δ (YKB4829), *yps5*Δ (YKB4830), *yps6*Δ (YKB4832), and *yps7*Δ (YKB4831) strains were grown to log phase in YPD, washed in YP and diluted to a final OD_600_ of 0.2. Four 10-fold serial dilutions were spotted onto YPD and YPM agar plates. (C) WT (YKB1079), along with *yps1*Δ, *yps3*Δ, *yps7*Δ, *yps1*Δ*yps7*Δ (YKB4897), *yps3*Δ*yps7*Δ (YKB4898), *yps1*Δ*yps3*Δ (YKB4899), and *yps1*Δ*yps3*Δ*yps7*Δ (YKB4900) strains were grown to log phase in YPD, washed in YP and diluted to a final OD_600_ of 0.2. Four 10-fold serial dilutions were spotted onto YPD and YPM agar plates. All plates were incubated for 2 days at 30° and are representative of three biological replicates.

It is known that *C. albicans* possesses SAPs that can break down mucin in growth media (Colina *et al.* 1996; De Repentigny *et al.* 2000), but it is not known if *S. cerevisiae* has proteins with similar functions. *C. albicans* Sap8 and Sap9 have the highest peptide sequence similarity to *S. cerevisiae* yapsin protein 1 (Yps1), while *C. albicans* Sap1, Sap2, Sap5, Sap6, Sap7, and Sap10 have the highest peptide sequence similarity to *S. cerevisiae* yapsin protein 3 (Yps3). These yapsins are part of a family of six aspartyl proteases that also include Yps2, Yps5, Yps6, and Yps7, many of which remain uncharacterized. Sap2 activity is induced upon mucin exposure (Colina *et al.* 1996), and our own transcriptome analysis found *YPS6* and *YPS7* were upregulated 1.3-fold and 1.5-fold, respectively (Supplementary File S1). Thus, we investigated the levels of gene expression of the *YPS* genes in mucin culture after 24 h of growth. Through qRT-PCR, both *YPS3* and *YPS7* were significantly induced 1.6-fold and 4-fold in YPM, respectively (Figure 6A). In parallel, we assessed the importance of individual yapsins for growth on mucin medium by conducting dot assays on YPD and YPM using deletion mutants for each of the six *YPS* genes (Figure 6B). While the majority of *yps* deletion mutants had no impact on growth when compared to WT cells, *yps7*Δ cells displayed modest but reproducible growth deficiency on YPM compared to WT cells.

As yapsin family members could be functionally redundant, we constructed double and triple deletion mutants with genes that had either high similarity to *C. albicans* SAPs (*yps1*Δ and *yps3*Δ) or growth defects on mucin medium (*yps7*Δ). While the *yps1*Δ*yps7*Δ and *yps3*Δ*yps7*Δ displayed mild growth defects similar to *yps7*Δ, the triple deletion mutant (*yps1*Δ*yps3*Δ*yps7*Δ) had reduced growth on YPD and a severe growth defect on YPM (Figure 6C). This suggests that Yps1, Yps3, and Yps7 are functionally redundant for a yet to be identified biological function. The fourfold upregulation of *YPS7* mRNA upon mucin exposure and the observable growth defect of *yps7*Δ cells suggest that Yps7 is important for *S. cerevisiae* growth in mucin media, and in its absence Yps1 and Yps3 can partially rescue growth in mucin conditions.

### Mucin induces cell wall stress but *yps7*Δ growth deficiency is not rescued with sorbitol

**Figure 7:**
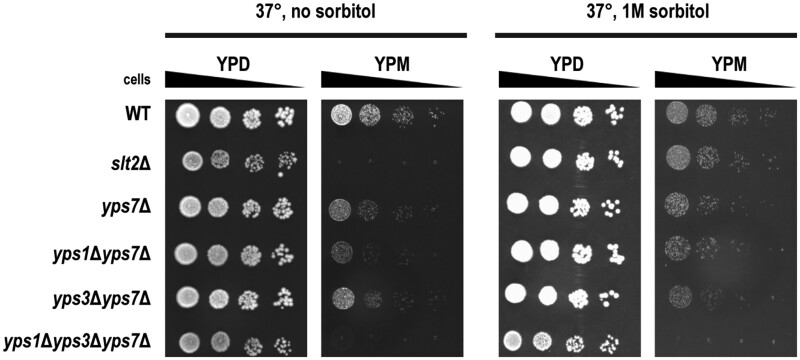
Mucin induces cell wall stress, but *yps1*Δ*yps3*Δ*yps7*Δ mucin sensitivity is not rescued by sorbitol. WT (YKB1079), along with *slt2*Δ (YKB4917), *yps7*Δ, *yps1*Δ*yps7*Δ, *yps3*Δ*yps7*Δ, and *yps1*Δ*yps3*Δ*yps7*Δ were grown to log phase in YPD, washed in YP and diluted to a final OD_600_ of 0.2. Four 10-fold serial dilutions were spotted onto YPD and YPM agar plates, with or without the addition of 1 M sorbitol. Plates were incubated for 3 days at 37° and are representative of three biological replicates.

Although there is little information known about the biological function of Yps7, previous research suggests it may be involved in cell wall integrity (Tong *et al.* 2004; Krysan *et al.* 2005). Therefore, we sought to determine whether mucin has an impact on cell wall integrity and if Yps7 is protective. As the osmotic stabilizer sorbitol rescues most mutants with cell wall defects at elevated temperatures (Martín *et al.* 1996), we asked if sorbitol could rescue the growth defects of *yps7*Δ, *yps1*Δ*yps7*Δ, *yps3*Δ*yps7*Δ and *yps1*Δ*yps3*Δ*yps7*Δ cells on YPM at 37°. To ensure sorbitol rescue for temperature-sensitive deletion mutants deficient in cell wall integrity, *slt2*Δ cells were used as a control since *SLT2* encodes a mitogen-activated protein kinase that regulates cell wall integrity (Martín *et al.* 1996). As expected, *slt2*Δ cells displayed mild but reproducible growth defects at 37° on YPD that was rescued by sorbitol (Figure 7). Surprisingly, *slt2*Δ cells did not grow on YPM at 37° and sorbitol rescued the growth defect, suggesting that mucin treatment is affecting cell wall integrity. In addition, while at 30° there was no observable genetic interaction (Figure 6C), *yps1*Δ*yps7*Δ displayed a mild growth defect at 37° on YPM. In contrast to the *slt2Δ* cells, sorbitol did not rescue the growth defects of *yps1*Δ*yps7*Δ or *yps1*Δ*yps3*Δ*yps7*Δ cells at 37° on YPM. Together, this suggests that mucin induces a cell wall stress, but the role of Yps1, Yps3, and Yps7 for growth on YPM is likely independent of cell wall processes.

## Discussion

Here, we use a variety of methods to determine that the laboratory *S. cerevisiae* strain S288C can grow in media with mucin as the main carbon source. Through transcriptome and chemical genomics screens, we uncovered a role for the mitochondria for growth on mucin media and determined that the uncharacterized protein Ycr095w-a is required for full mitochondrial function under mucin conditions. Similar to *C. albicans*, our work also identified the importance of the aspartyl protease Yps7 for growth on mucin or limited carbon sources. Importantly, this work suggests that *S. cerevisiae* has the potential to colonize the mucin-enriched gut environment.

### Impact of mucin on cell growth and morphology

Multiple pieces of evidence presented here indicate that like *C. albicans*, *S. cerevisiae* cells are also able to utilize mucin as a carbon source, including increased growth and OCRs when cultured in the presence of mucin compared to in the absence of an added carbon source (Figures 1, B and C, and 5). Though cells grown in poor carbon sources have smaller critical sizes before cells progress out of G1 phase (Johnston *et al.* 1979), we were surprised that mucin cultured cells were smaller than cells grown in medium with no added carbon source (Figure 1E). This suggests that progression out of G1 phase may occur faster due to a lower critical size requirement in mucin conditions. Importantly, cells in mucin culture reach this reduced size by 24 h and still continue to progress through the cell cycle, although at a slower rate. Alternatively, but not mutually exclusive, the reduction in cell size could reflect adaptation to hyper-osmotic stress induced by mucin glycoproteins in the medium. Exposure to a hyper-osmotic stress, such as high sugar or salt concentrations, results in activation of the high osmolarity glycerol (HOG) pathway, rapid loss of water, cell shrinkage and cell cycle arrest prior to adaptation (Saito and Posas 2012). In agreement with the possibility that mucin exposure can activate the HOG pathway, our transcriptome analysis revealed that *STE20*, which encodes a p21-activated kinase (Roberts and Fink 1994) and is an upstream activator of the Sho1 branch of the HOG pathway, was upregulated fourfold in mucin culture (Table 2). Conversely *SKN7*, which encodes a key regulator of cell wall biogenesis and the hypo-osmotic stress response (Fassler and West 2011), was downregulated by ninefold (Table 3). Though Skn7 does not directly inhibit the HOG pathway, Skn7 opposes the function of the HOG pathway, fine-tuning cell size after hyper-osmotic stress through both the cell wall integrity and hypo-osmotic stress response pathways (Fassler and West 2011; Saito and Posas 2012). Hence, the reduction in cell size may be an adaptive response to mucin conditions regulated by the HOG pathway. It will be interesting to determine if clinical isolates of *S. cerevisiae* undergo morphological or cell size changes upon mucin exposure and if this adaptive response to mucin exposure is required for colonization within gut niches.

### Impact of mucin on mitochondrial morphology and function

Exactly how mucin glycoproteins are broken down and used by *S. cerevisiae* is still speculative. However, our work suggests that yeast consider mucin a poor carbon source and require mitochondria and cellular respiration for growth in mucin conditions. Under carbon-limiting conditions, mitochondria typically undergo fission (Visser *et al.* 1995) and mitochondrial biogenesis (Priault *et al.* 2005), including the induction of genes in mitochondrial processes like the tricarboxylic acid cycle and oxidative phosphorylation (Boer *et al.* 2003; Wu *et al.* 2004). In our mucin transcriptome study, 11 of the top 50 most upregulated genes are associated to the mitochondrion (Table 2), including ATP synthase biogenesis (*ATP22*, *ATP23*, and *OXA1*) and a key regulator of mitochondrial fission (*FIS1*) (Mozdy *et al.* 2000). Similar to yeast grown in ethanol or aerobic, glucose-limiting chemostat cultures (Visser *et al.* 1995), WT cells displayed both an increase in the mitochondrial marker Cit1-RFP signal and the formation of short mitochondrial structures dispersed throughout the cell in mucin medium (Figure 4A). Furthermore, OCR measurements confirmed that mucin treatment resulted in increased mitochondrial function, even more so than cells grown in ethanol (Figure 5). Together, this provides evidence that oxidative phosphorylation and the proper functioning of the electron transport chain are important for growth in mucin conditions.

Due to the presence of a carbon source, even as complex as mucin, it was expected that there would be mitochondrial-associated genes that when deleted, resulted in worse growth in the presence of mucin (YPM) compared to media with no added carbon source (YP). From the chemogenomic screen, *CCM1* and *YCR095W-A* deletion mutants fit this profile (Figure 3B). Though deletion mutants of *CCM1* or *YCR095W-A* displayed similar fragmented mitochondrial morphology in YPM as WT cells (Figure 4A), there was a significant increase in Cit1-RFP signal in both mutants compared to WT cells (Figure 4B). Given that the mitochondria of *ccm1*Δ cells are nonfunctional (Figure 5), the increase in Cit1-RFP signal seen in mucin culture may reflect a cellular feedback-loop to increase mitochondrial activity. Interestingly, while deletion of *YCR095W-A* did not significantly impact OCR under YP or YPE, there was a significant decrease in OCR in mucin (Figure 5), indicating that Ycr095w-a is required for oxygen consumption within mucin medium. No previous work has been conducted on this putative gene, and its peptide sequence bears no resemblance to other proteins in *S. cerevisiae* S288C nor other yeast species. The characterization of this protein, both in its structure and localization, could help determine a functional role during mucin metabolism and overall stress in mucin or other carbon-limiting conditions.

### The importance of yapsins for growth in mucin conditions

Our study is the first to demonstrate that SAP homologs found in *S. cerevisiae* (yapsins) also have a role in the cellular response to mucin. We showed that *YPS7* mRNA was upregulated in mucin conditions (Figure 6A and Supplementary File S1) and cells in which *YPS7* was deleted compromised growth on mucin (Figure 6, B and C). While no other single yapsin mutant displayed growth defects on mucin, our work suggests that Yps1 and Yps3 can compensate for the absence of *YPS7* on mucin (Figures 6 and 7).

What are yapsins and why are they needed for growth on mucin? Presently, one of the few known roles for the *S. cerevisiae* yapsin family is for cell wall integrity. Yapsins were shown to be induced concomitantly in conditions that stimulate the expression of cell wall genes (Bourbonnais *et al.* 2001). Furthermore, Krysan *et al.* (2005) demonstrated that deletion of *YPS1* had the most growth deficiency upon treatment with the cell wall stressors Congo Red (disrupts chitin/β-glucan fibril formation) and caffeine (causes general cell wall stress at 37^°^), while deletion of *YPS7* had a specific growth deficiency upon treatment with calcofluor white (disrupts chitin polymer formation). Our study demonstrates that mucin exposure can induce cell wall stress as the growth defect of *slt2*Δ cells on mucin was rescued by sorbitol (Figure 7). It is known that Yps1 levels increase 12-fold in response to a temperature shift from 24° to 37° (Ash *et al.* 1995) and Yps1 has been implicated in the MAPK pathway (Vadaie *et al.* 2008). However, the growth deficiency on YPM at 37° observed in *yps7*Δ, *yps3*Δ*yps7*Δ and *yps1*Δ*yps3*Δ*yps7*Δ mutants was not rescued upon the addition of 1 M sorbitol. Any mucin impact on cell wall integrity does not appear to be the main factor for growth deficiency on mucin medium in *yps7*Δ mutants.

In summation, this study demonstrates that even laboratory *S. cerevisiae* strains can grow in media with mucin as the main carbon source via modulating mitochondrial dynamics and the conserved importance of fungal aspartyl proteases for growth in mucin. Our work supports that *S. cerevisiae* has the potential of being a gut colonizer as opposed to just an environmental organism passing through the gastrointestinal tract, and complements recent research showing that *S. cerevisiae* represents a major player in the inter-kingdom dynamics of the gut microbiome (Roussel *et al.* 2018; Sovran *et al.* 2018). By understanding how the most common dietary fungus can live in a mucin environment, we can further our knowledge on the microbial functional interactions that impact human health.

## Data availability

Strains are available upon request. Supplementary File S1 contains the complete RNA-seq and gene functional classification. Supplementary File S2 contains the mucin chemogenomic screen datasets. Raw sequencing reads have been deposited at the NCBI SRA archive under accession number PRJNA679297.

Supplementary material available online at *G3*.

## Supplementary Material

jkab294_Supplementary_Data
