# Supplementary material for: Global analysis of Saccharomyces cerevisiae growth in mucin
Source: G3 (Bethesda). 2021 Aug 18;11(11):jkab294. doi: 10.1093/g3journal/jkab294 (PMC8527512; doi:10.1093/g3journal/jkab294)
Supplement: jkab294_Supplementary_Data [file jkab294_supplementary_data.zip › GENETICS-G3-2021-402654-s05.docx]

**Figure S1** SK1 cells form pseudohyphae when cultured in mucin media. Live-cell imaging of *S. cerevisiae* SK1 cells grown in different media. SK1 cells were grown to log phase in YPD, washed in YP and reinoculated into 50 mL of YPD, YP and YPM media and grown at 30° for 12 hrs or 24 hrs prior to brightfield imaging. Scale bar represents 10 μm.

**Figure S2** Principal component analysis (PCA) and transcriptional profiling demonstrate separation by sample treatment and a large set of differentially expressed genes. (A) PCA of normalized gene counts of the first two principal components shows separation of YPM samples (red) and YP samples (green). (B) Volcano plot for all 739 differentially expressed genes from RNA sequencing experiments. Thresholds shown here are for differential expression levels of aligned sequences calculated at fold changes ≥ 2, and a FDR adjusted p-value ≤ 0.01 (red dots).
