# Supplementary figures and images for: Global analysis of Saccharomyces cerevisiae growth in mucin
Source: G3 (Bethesda). 2021 Aug 18;11(11):jkab294. doi: 10.1093/g3journal/jkab294 (PMC8527512; doi:10.1093/g3journal/jkab294)

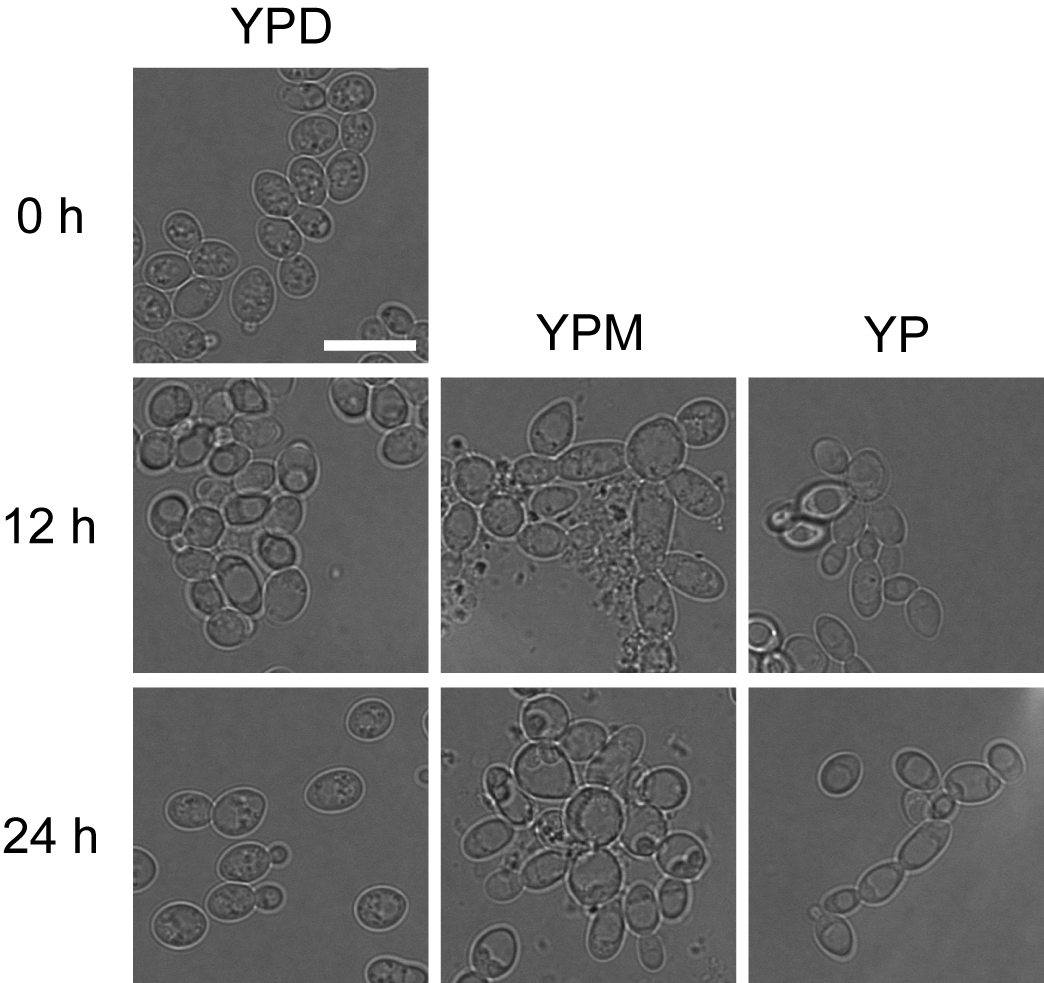

Supplement: jkab294_Supplementary_Data [file jkab294_supplementary_data.zip › GENETICS-G3-2021-402654-s01.tif]

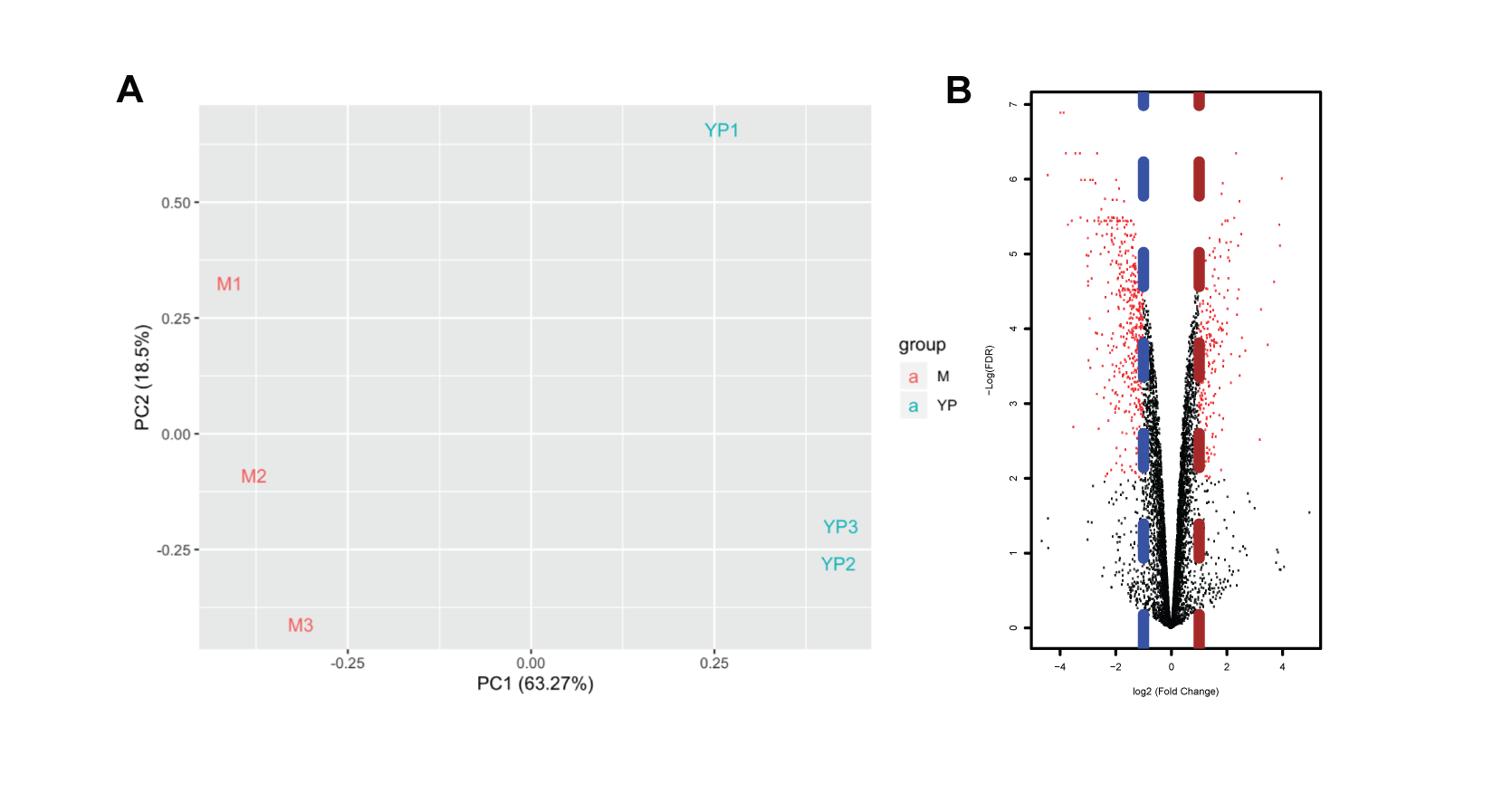

Supplement: jkab294_Supplementary_Data [file jkab294_supplementary_data.zip › GENETICS-G3-2021-402654-s02.tif]
